# Revolutionizing cancer treatment: the emerging potential and potential challenges of *in vivo* self-processed CAR cell therapy

**DOI:** 10.7150/thno.101941

**Published:** 2024-10-28

**Authors:** Ruijie Lv, Yanting Guo, Weici Liu, Guangjian Dong, Xiangyin Liu, Caihui Li, Yi Ren, Zipeng Zhang, Shi-Yong Neo, Wenjun Mao, Jing Wu

**Affiliations:** 1 Department of Pharmacy, The First Affiliated Hospital of Shandong First Medical University & Shandong Provincial Qianfoshan Hospital; Jinan 250014, China; School of Pharmacy, Shandong First Medical University & Shandong Academy of Medical Sciences, Jinan, Shandong 250117, China.; 2 Department of Thoracic Surgery, The Affiliated Wuxi People's Hospital of Nanjing Medical University, Wuxi People's Hospital, Wuxi Medical Center, Nanjing Medical University, Wuxi, Jiangsu 214023, China.; 3 Department of Clinical Pharmacy, School of Pharmacy, Shandong Second Medical University, Weifang, Shandong 261042, China.; 4 Medical Science and Technology Innovation Center Shandong First Medical University & Shandong Academy of Medical Sciences Jinan 250117, China.; 5 Singapore Immunology Network, Agency for Science, Technology and Research, Singapore 138648, Republic of Singapore.

**Keywords:** CAR, Immunotherapy, Construction, Delivery technology, Vector particles

## Abstract

Chimeric antigen receptor (CAR) cell immunotherapies, including CAR-T, CAR-Macrophages, CAR-Natural Killer, CAR-γδ T, etc., have demonstrated significant advancements in the treatment of both hematologic malignancies and solid tumors. Despite the notable successes of traditional CAR cell manufacturing, its application remains constrained by the complicated production process and expensive costs. Consequently, efforts are focused on streamlining CAR cell production to enhance efficacy and accessibility. Among numerous proposed strategies, direct *in vivo* generation of CAR cells represents the most substantial technical challenge, yet holding great promise for achieving clinical efficacy. Herein, we outlined the current state-of-the-art *in vivo* CAR therapy, including CAR technology development, transfection vectors, and influence factors of construction of CAR *in vivo*. We also reviewed the types and characteristics of different delivery systems and summarized the advantages of *in vivo* CAR cell therapy, such as rapid preparation and cost-effectiveness. Finally, we discussed the limitations, including technical issues, challenges in target and signal design, and cell-related constraints. Meanwhile, strategies have correspondingly been proposed to advance the development of CAR cell therapy, in order to open the new horizons on cancer treatment.

## 1. Introduction

Immunotherapy has transformed therapeutic approaches, establishing itself as the fourth pillar of cancer treatment, alongside surgery, radiotherapy, and chemotherapy 1. It has significantly improved the prognosis for metastatic cancer, offering long-term remissions and even potential cures for patients. In 2023, immunotherapy, including ICIs and CAR-T cell therapies, continued to improve cancer treatment outcomes. ICIs raised the five-year survival rate for advanced lung cancer to 23%, up from 5% 2. CAR-T cell therapy showed a 58% survival rate in patients with large B-cell lymphoma, with 41% achieving long-term remission 3. Immunotherapy focuses on various components of the immune system, employing strategies such as tumor-infiltrating lymphocytes (TIL), CAR T cells, CAR natural killer (NK) cells, and T cell receptors 4,5.

CAR cell therapy is an immunotherapy that utilizes genetically engineered immune cells to target and destroy both cancerous and certain non-cancerous cells. Over 700 clinical trials are currently evaluating the efficacy of CAR-T cell therapy in solid tumors, as listed on clinicaltrials.gov 6. CAR-Macrophages (CAR-M) cell therapy leverages the innate properties of macrophages, equipping them with specific anti-tumor functions. Similarly, NK cells can recognize and eliminate cancer cells without prior activation 7,8, and this capability is further enhanced in CAR-NK cell therapy, which aims to improve therapeutic outcomes by more precisely targeting cancer cells. Notably, NK cells secrete a diverse array of cytokines, contributing to robust anti-tumor immunity 9. While different CAR cell therapies share key features, such as the use of genetic engineering to target specific cancer antigens and initiate immune activation and cancer cell elimination, they vary in the type of immune cells used, the cancers they target, their mechanisms of action, persistence, and risk of graft-versus-host disease (GvHD). For instance, CAR-T cell therapy is primarily used for hematologic malignancies, providing long-lasting effects but posing a higher risk of GvHD. In contrast, CAR-NK cell therapy is effective against both hematologic and solid tumors, with a lower risk of adverse toxicity effects but still limited by poor persistency *in vivo*. CAR-M cell therapy excels homing into solid tumors and reshaping the immune microenvironment 10.

**Figure 1:**
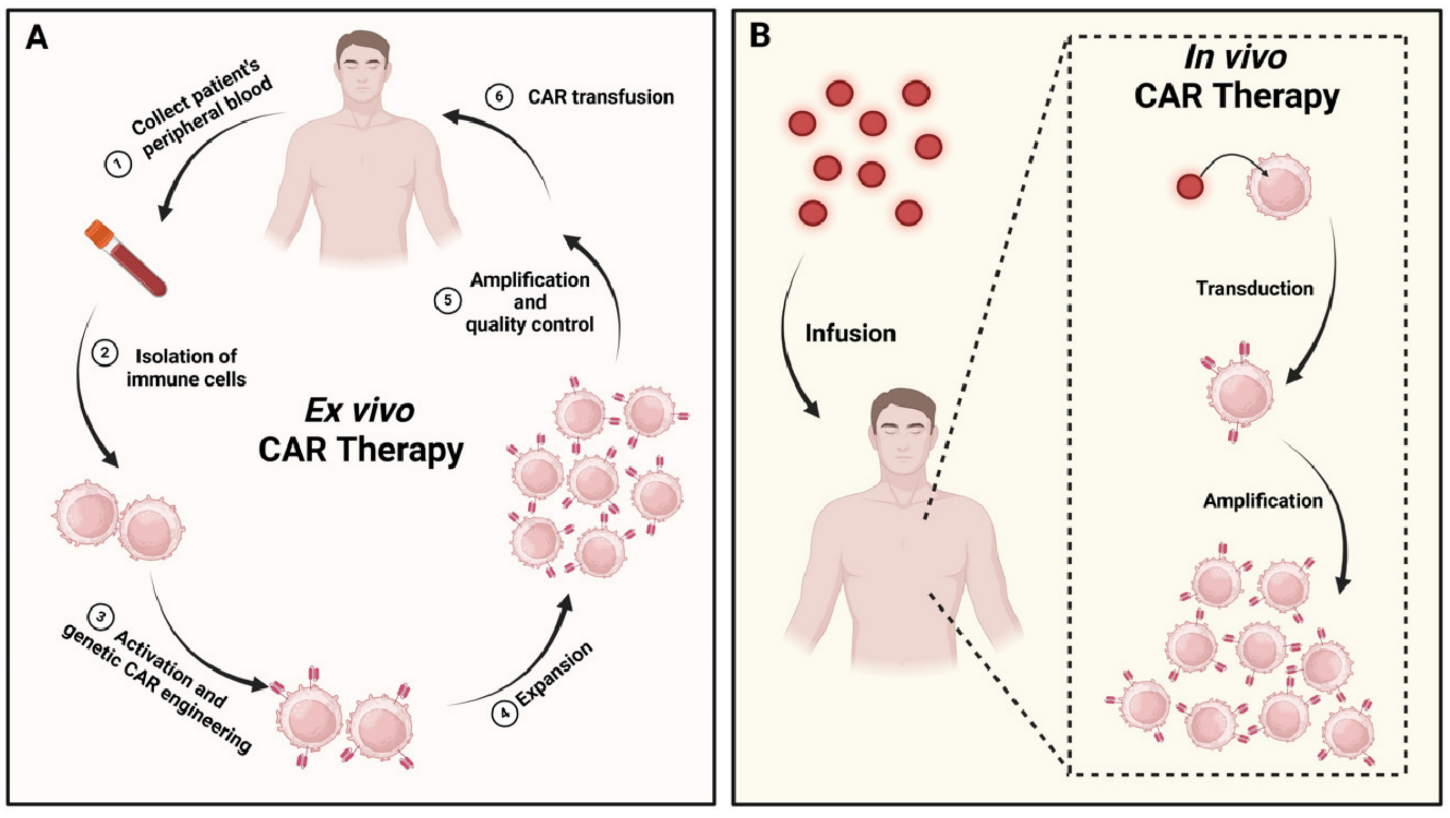
***Ex vivo* vs.* in vivo* CAR cell therapies**: **a comparative.** (A) The *ex vivo* approach begins with the isolation of immune cells from the patient's blood. These cells are then activated, expanded, and genetically modified in a controlled laboratory environment. Following stringent quality control measures, the engineered CAR cells are reinfused into the patient. (B) In contrast, the *in vivo* method involves directly infusing vector particles (represented as red dots) into the patient. These vectors interact with the patient's immune cells within the body, selectively transferring the genetic material necessary to encode the CAR.

Despite the promising advances, CAR cell therapies face significant challenges due to the personalized cell engineering and manufacturing processes required. These processes are essential but extend the treatment timeline to several weeks or even months. The delays, combined with the high costs of cell engineering, production, and treatment monitoring, make CAR cell therapies prohibitively expensive for widespread use. An emerging solution to these challenges is the *in vivo* construction of CAR cells. This approach circumvents the time-consuming *ex vivo* engineering process 11, simplifies treatment by reducing the number of complex steps involved, and lowers overall costs, making it more economically feasible compared to* ex vivo* construction (**Figure 1**) 12. While the development of CAR cell therapies presents a promising avenue for cancer treatment, innovative strategies are needed to overcome the existing technical and economic barriers. This review article provides an overview of the structure and evolution of CARs, various types of CAR cell therapies, key vector technologies used in CAR cell therapy delivery and factors influencing CAR construction.

## 2. Generation and development of CAR technology

CAR cell therapy marks a significant leap in cancer treatment, evolving from earlier TCR-based therapies, which were limited by Human Leukocyte Antigen HLA restrictions and tumor escape mechanisms 13,14. To overcome these challenges, CARs were engineered by combining functional components, leading to advancements in CAR cell therapy 15. Initially, CARs had a simple structure with an antigen-binding domain and a signal transduction domain. Over time, improvements such as adding costimulatory molecules and refining signal strength have greatly enhanced their efficacy 16. Today, CAR cell therapy includes NK cells and macrophages, enabling personalized treatments for various cancers 17,18.

### 2.1. The structure of CAR

The modular design of CARs includes four key components: the antigen-binding domain, hinge, transmembrane domain, and intracellular signaling domain 19. Each part plays a distinct role, allowing for optimal design flexibility. The antigen-binding domain, typically a single-chain variable fragment (scFv) made from the variable regions of monoclonal antibodies, is critical for recognizing and binding tumor antigens 20. The (Gly4Ser)3 linker, commonly used to connect antibody fragments, ensures proper folding and antigen binding 21. This precision enhances CARs' ability to target cancer cells, significantly improving therapeutic outcomes 22. The hinge and transmembrane domains connect the extracellular and intracellular components of the CAR. The hinge provides flexibility to avoid steric hindrance, aiding antigen capture near the membrane 23. Hinge domains often include sequences from CD8, CD28, IgG1, or IgG4 24. The transmembrane domain, commonly derived from proteins like CD3ζ, CD28, or CD8α, anchors the CAR, ensuring stability and function 25. The intracellular signaling domain contains an activation domain, usually derived from CD3ζ, and costimulatory domains from molecules like CD28 or 4-1BB, which are critical for effective CAR activation and have received FDA approval 26,27. The CAR structure used in CAR-T cell therapy mirrors those in CAR-NK and CAR-M cell therapies, all of which combine antigen recognition, transmembrane anchoring, and intracellular signaling to enable precise tumor targeting and elimination by genetically modified immune cells.

### 2.2. The development process of CAR cells

**Figure 2:**
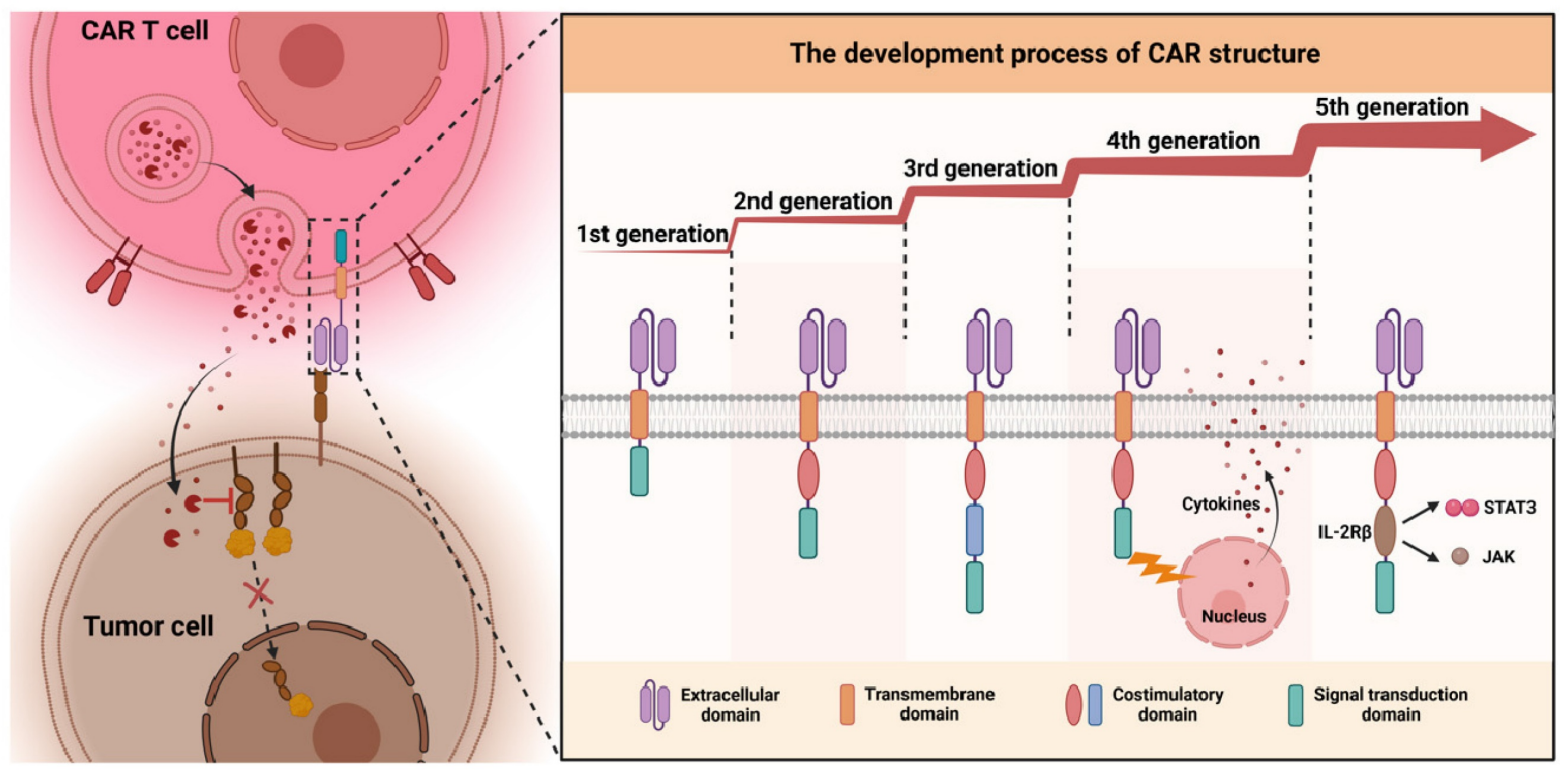
** The development process of CAR cells from the first to the fifth generation:** CAR cells are categorized into five generations, each defined by distinct intracellular signal transduction structures.

CAR cells are currently classified into five generations, each distinguished by its intracellular signaling structures (**Figure 2**) 28-31. The first generation introduced a basic CAR configuration, featuring an extracellular antigen recognition domain from the variable region of a monoclonal antibody, and an intracellular signaling domain that typically utilizes the CD3ζ chain 32. This design enables CARs to recognize tumor antigens and trigger an immune response through CD3ζ signaling 33,34, laying the foundation for genetically engineered immune cells to target and destroy tumor cells 35.

The second generation of CAR cells introduced costimulatory molecules like CD28 or 4-1BB alongside the CD3ζ chain, enhancing cell survival, proliferation, and tumor-killing ability 36. Carl *et al.* developed a second-generation CAR-T targeting CD19 with a 4-1BB costimulatory domain, leading to CTL-019, the first FDA-approved CAR-T product 37. The third generation incorporated additional costimulatory molecules, such as OX40 and ICOS, along with CD28 or 4-1BB, to boost CAR cell activation and persistence, but results have been mixed, with some studies showing no significant improvements over the second generation 38-41. The fourth generation, called “combined antigen receptors” or TRUCKs (T cells Redirected for Universal Cytokine Killing), retains traditional CAR functions but adds the ability to secrete specific cytokines, such as IL-12. This helps modulate the tumor microenvironment, enhancing anti-tumor efficacy by recruiting and activating other immune cells like NK cells and macrophages 42. The fifth generation, referred to as “Universal CAR,” features an optimized design with a tumor antigen recognition region, a costimulatory signaling region, and an enhanced signaling domain. This allows for better CAR cell activation and persistence, crucial for overcoming tumor microenvironment challenges and improving tumor cell eradication 43,44. Each generation builds upon the last, optimizing CAR technology for improved efficacy and expanding treatment possibilities for cancer patients.

In summary, the progressive evolution of CAR structures has primarily focused on augmenting cell activity, persistence, and functionality while improving their performance within the challenging tumor microenvironment. Each generational innovation has aimed to resolve the limitations of its predecessor, gradually enhancing the clinical potential and applicability of CAR cell therapy.

### 2.3. Different CAR cell therapies

**Figure 3:**
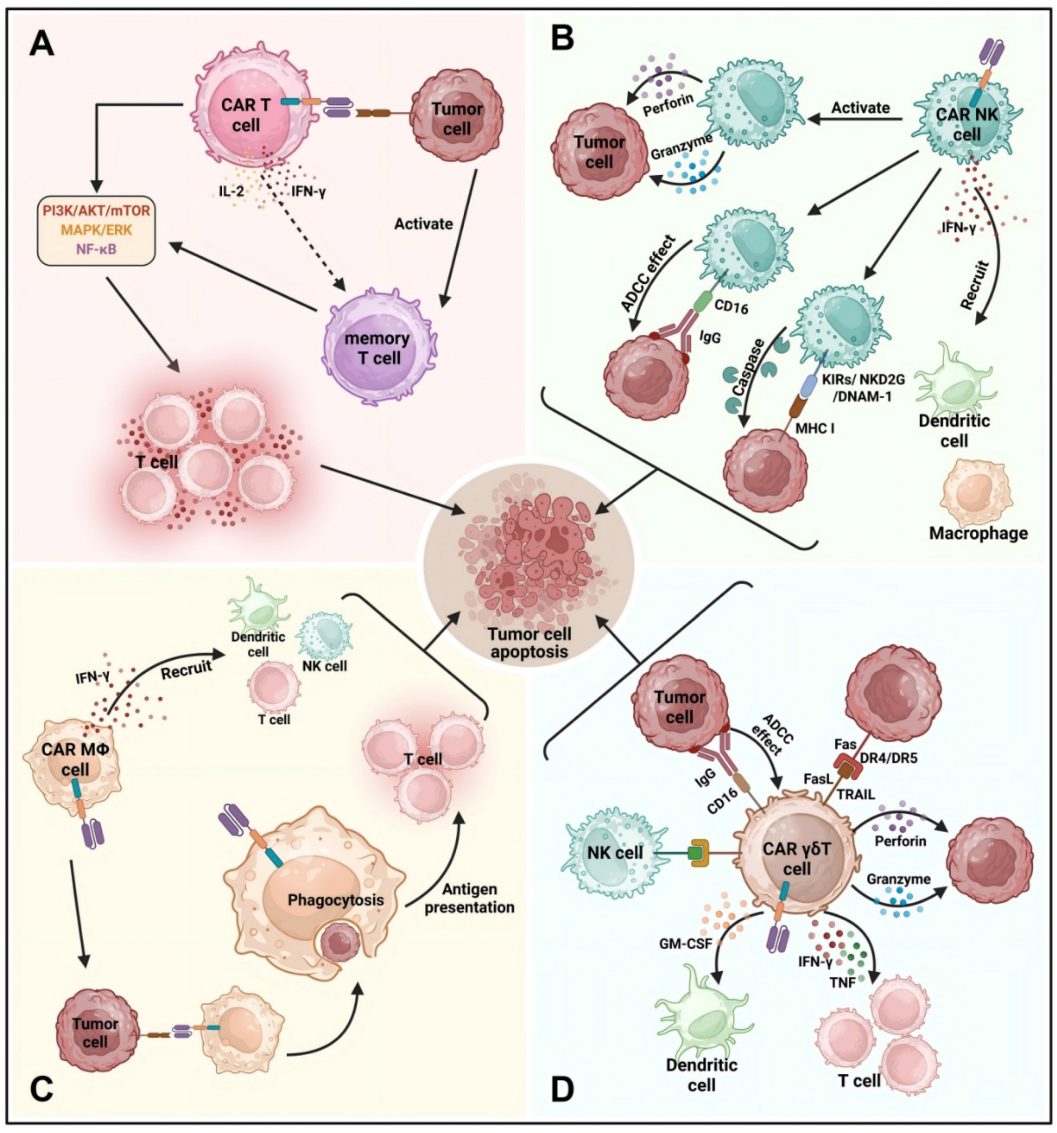
** The mechanisms of CAR-T, CAR-NK, CAR-M, and CAR-γδ T cell therapies.** (A) When CAR-T cells bind to target antigens, they activate intracellular signaling pathways like PI3K/AKT/mTOR, MAPK/ERK, and NF-κB, leading to T cell proliferation, cytokine production, and cytotoxic activity through perforin and granzyme release, inducing cancer cell apoptosis. CAR-T cells also secrete cytokines such as IFN-γ and IL-2, boosting the antitumor response, with some differentiating into memory T cells for long-term immune surveillance and reduced cancer recurrence. (B) Similarly, activated CAR-NK cells produce perforin and granzyme, promoting cancer cell death through caspase-mediated apoptosis. CAR-NK cells express receptors such as KIRs, NKG2D, and DNAM-1, and mediate antibody-dependent cellular cytotoxicity (ADCC) via CD16, playing a crucial role in targeting HER2 and EGFR in solid tumors. Their efficient ADCC is linked to better outcomes in various cancers. (C) CAR-M cell therapy uses engineered macrophages to enhance phagocytosis and antigen presentation, adapting to tumor environments while promoting pro-inflammatory signaling and suppressing tumor-promoting polarization. CAR-M cells secrete IFN-γ, recruiting and activating immune cells, and upregulate MHC-I and MHC-II, improving T cell activation and immune infiltration into tumors. (D) CAR-γδ T cells exhibit antitumor effects via TCR, NKRs, and CD16, triggering ADCC and directly killing tumor cells by releasing TRAIL, FasL, perforin, and granzyme. They enhance cytotoxic T cell and NK cell functions through IFN-γ, TNF, and CD137 signaling, and produce GM-CSF to regulate dendritic cell infiltration, further augmenting the antitumor immune response.

In this review, we briefly outline the mechanisms and application of CAR-T, CAR-NK, CAR-M, and CAR-γδ T cell therapies (**Figure 3**).

#### 2.3.1 CAR-T cell therapy

CAR technology, initially developed for T cells, has revolutionized immunotherapy by enhancing its synergy with other immune cells 45. Over time, CAR cell therapy expanded to various immune cells, offering new medical possibilities. The process involves creating a recombinant plasmid that merges an antibody fragment targeting tumor antigens with an immune receptor tyrosine activation motif (ITAM). This plasmid is transfected into patient T cells, enabling them to target tumor cells 46. CAR-T cell therapy has been highly effective in treating cancers like leukemia, lymphoma, and glioma 47,48. The treatment involves extracting, modifying, and reinfusing T cells, which then target cancer cells. Once the CAR-T cells bind to the tumor antigen, they activate pathways such as PI3K/AKT/mTOR, leading to T cell proliferation, cytokine release, and cytotoxic activity. Cytokines like IFN-γ and IL-15 enhance the immune response, and memory T cells provide long-term protection, reducing cancer recurrence risks 49-51. Although no CAR-T cell products have been approved for the treatment of solid tumors, numerous clinical trials are underway exploring their application in this area. The following targets are currently being investigated (**Table 1**).

As of now, six CAR-T cell therapies have received FDA approval, three of which target B-cell maturation antigen (BCMA), while the others target CD19 (**Table 2**). Despite significant breakthroughs in tumor treatment, CAR-T cell therapy faces several challenges, such as the high cost and complex manufacturing process ahead of widespread clinical application, severe adverse reactions in company with CAR-T cell therapy, and insufficient response and even resistance in certain applications.

#### 2.3.2 CAR-NK cell therapy

NK cells are crucial to the innate immune system, able to eliminate tumor cells without prior sensitization 52. Both autologous and allogeneic NK cell infusions show promise in treating relapsed/refractory acute myeloid leukemia (AML) 53. CAR-NK cells combine CAR's precision with NK cells' innate tumor-killing ability, improving targeting of AML 54. They offer a favorable safety profile with lower risks of cytokine release syndrome (CRS) and neurotoxicity. NK cells also express less PD-1, reducing immunosuppression in the tumor environment 55,56. CAR-NK cells have lower immunogenicity and reduce the risk of graft-versus-host disease (GVHD), while maintaining durable antitumor effects.

NK cells naturally kill tumor cells using cytotoxic molecules like perforin and granzyme, without needing antigen recognition like T cells 57,58. They use receptors like NKG2D and DNAM-1 to trigger cancer cell death, and higher expression of these receptors improves cancer outcomes 59,60. NK cells also help immune responses by releasing IFN-γ and mediate antibody-dependent cellular cytotoxicity (ADCC) through CD16 receptors, aiding in therapies against HER2 and EGFR in solid tumors 61,62.

CAR-NK cells enhance NK cell activity by targeting specific cancer antigens, using CAR constructs with NK-specific domains like NKG2D and DAP-10 to increase cytotoxicity and cytokine production 63. Despite the challenges posed by the tumor microenvironment, CAR-NK therapy shows promise in clinical trials 64. There have been over 20 reported clinical trials involving CAR-NK cells, three of which have been fully completed (**Table 3**). Combining CAR-NK cell therapy with other treatments could improve outcomes by overcoming immune suppression in tumors 65. In tumor immunotherapy, particularly for solid tumors, both preclinical and clinical research on CAR-NK cells urgently require the development of more efficient infection methods and safer non-viral transfection technologies to overcome the inhibitory tumor microenvironment and continuously accumulate clinical experience. By improving infection techniques, CAR-NK cells can be more efficiently engineered to express CARs, enhancing their ability to target and destroy tumor cells. Efficient infection methods ensure a higher transduction rate, equipping more NK cells with the CARs needed to recognize and kill cancer cells, even within the suppressive tumor environment. Furthermore, non-viral transfection methods, such as electroporation or nanoparticle delivery systems, offer a safer alternative by reducing risks associated with viral vectors, such as insertional mutagenesis and immune responses. This approach enables repeated dosing or modification of NK cells without the drawbacks of viral methods, promoting their persistence and functionality in the tumor microenvironment 66.

#### 2.3.3 CAR-M cell therapy

CAR-M cell therapy employs genetically engineered macrophages to enhance their phagocytic capacity and improve antigen presentation to tumor cells. This approach not only boosts antigen presentation, thereby increasing T cell cytotoxicity and facilitating tumor cell engulfment, but it also adapts to environmental signals, potentially modifying its phenotype. Compared to CAR-T cell therapy, CAR-M therapy presents advantages such as reduced off-target toxicity and shorter treatment durations. Several CAR-M cell therapy candidates are currently in different phases of preclinical and clinical research and development (**Table 4**). CAR recognition of tumor antigens initiates macrophage-mediated antibody-dependent cellular cytotoxicity (ADCC) 67. This process involves antibodies against carcinoembryonic antigen (CEA) on macrophages binding to the Fc region of CEA antibodies on cancer cells, thereby inducing antibody-dependent cellular cytotoxicity (ADCC) to eliminate CEA-expressing tumor cells. Upon binding to specific tumor antigens, CAR receptors activate intracellular signaling pathways that promote phagocytosis, directly killing cancer cells and facilitating rapid antigen presentation to activate T cell-mediated immunity 68. CAR-mediated signaling prevents macrophage polarization towards a tumorigenic phenotype while activating pro-inflammatory pathways. CAR-M cells secrete pro-inflammatory cytokines, such as interferon-gamma (IFN-γ), which recruit and activate other immune cells to further target cancer cells. As CAR-M cells eliminate more cancer cells, they stimulate adaptive immune responses, providing effective and durable antitumor immunity. CAR-M activation typically upregulates MHC-I and MHC-II expression, enhancing the presentation of tumor-associated antigens and promoting T cell activation. Furthermore, CAR-M cells enhance the infiltration of CD4+ and CD8+ T cells, NK cells, and dendritic cells within tumors, augmenting immune-mediated cytotoxicity against solid tumors 69.

Compared to CAR-T and CAR-NK, CAR-M cell therapies presents unique advantages as a novel cell-based immunotherapy 10. These advantages include the ability to establish a pro-inflammatory environment within the tumor and to reverse the immunosuppressive tumor microenvironment. In preclinical animal studies, CAR-M has demonstrated effective anti-tumor capabilities. However, the efficacy and safety of CAR-M cell therapy still require validation in clinical settings.

#### 2.3.4 CAR-γδ T cell therapy

In cell therapy, CAR-T cell therapy primarily targets αβ T cells, making up about 95% of the T cell population 70. In contrast, γδ T cells, which play a complex role in tumor immunology, exhibit significant antitumor activity, especially in humans 71,72. They activate through γδ T cell receptors (TCRs), natural killer receptors (NKRs), and CD16, enabling antibody-dependent cellular cytotoxicity (ADCC) against tumor cells 73. γδ T cells can directly eliminate tumors via TCR and NKR engagement and secrete cytotoxic granules containing perforin and granzymes.

Moreover, γδ T cells enhance antitumor responses by secreting IFN-γ and TNF, improving αβ T cell function and MHC I expression on tumor cells. They also stimulate NK cells and produce GM-CSF to regulate dendritic cell infiltration 74. However, γδ T cells can have protumor effects, particularly through IL-17, which promotes tumor growth and angiogenesis in certain contexts 75. IL-17+ γδ T cells have been observed in advanced cancer stages, contributing to immune evasion 76,77.

Engineered with CARs, γδ T cells can specifically target tumor antigens, kill tumor cells, and recruit other immune cells, making them promising for solid tumors due to their ability to penetrate the tumor microenvironment 78. Unlike the more common αβ T cells, γδ T cells, which constitute about 0.5%-5% of T cells, possess both innate and adaptive immune features, allowing for rapid recognition of non-MHC-restricted antigens 79,80. CAR-γδ T cell therapies show significant potential, although clinical trials are limited. For example, the phase I trial ADI-001 (NCT04735471) targeting CD20 for B-cell non-Hodgkin lymphoma has shown promising efficacy and safety 81. Compared to CAR-T cell therapy, CAR-γδ T cell immunotherapy offers advantages such as MHC-independent recognition, applicability to various tumor types, and reduced risk of cytokine release syndrome, highlighting their potential as a therapeutic approach 82.

#### 2.3.5 Other CAR cell therapies

In addition to CAR-T, CAR-NK, CAR-M, and CAR-γδ T cell therapies, several other CAR cell therapies are emerging. Chimeric Antigen Receptor Natural Killer T (CAR-NKT) cells involve genetically engineering NKT cells to express chimeric antibodies, allowing for targeted destruction of tumor cells while preserving their innate antitumor properties 7. This dual functionality enhances overall immune responses against tumors, offering long-term protection and improved persistence through interleukin 15 (IL-15) inclusion 64,83. Similarly, CAR-DC cell therapy utilizes the antigen-presenting capabilities of dendritic cells (DCs), modifying them with CARs to directly target cancer cells. This enhances T cell responses through effective antigen presentation, cytokine secretion, and recruitment of additional immune cells 84. In contrast, CAR-Treg cell therapy focuses on regulatory T cells (Tregs) engineered to recognize specific antigens, suppressing immune activation through mechanisms like CTLA-4 engagement with CD80 and the release of inhibitory cytokines 85. This approach is useful for preventing immune rejection and maintaining immune homeostasis. Lastly, Chimeric Autoantibody Receptor T (CAAR-T) cells are designed to selectively target autoreactive B cells by recognizing autoantibody proteins on their surface 86. This method effectively eliminates memory B cells and plasma cells that produce pathogenic autoantibodies without causing widespread immune suppression 87. CAAR-T therapy shows promise in treating autoimmune disorders like Pemphigus Vulgaris, with ongoing clinical trials assessing optimal dosing and efficacy 88.

Research on CAR-T cell therapies for solid tumors is still limited compared to their success in hematologic malignancies. Challenges include a lack of tumor-specific antigens, low CAR-T cell trafficking efficiency, and an immunosuppressive microenvironment. In contrast, CAR-NK and CAR-M cells offer advantages in treating solid tumors due to their strong antitumor properties and reduced side effects like cytokine release syndrome (CRS). However, issues such as "on-target, off-tumor" toxicity and antigen escape remain. CAR-M cell therapy combines innate and adaptive immune responses, enhancing tumor regression through interactions with T and NK cells. Combining CAR-M with CAR-NK or CAR-T cells could improve efficacy against various tumor antigens. Advanced technologies like artificial intelligence (AI) and radiomics are being utilized to optimize CAR cell therapies, with AI predicting new cancer-associated antigens and improving CAR-T cell manufacturing, while radiomics provides insights into the tumor microenvironment. Additionally, CAR cell therapies are being investigated for autoimmune diseases. For instance, CAR-T cells targeting CD19 have shown promise in lupus nephritis, and CAR-Tregs have been effective in multiple sclerosis and type 1 diabetes by suppressing immune attacks while maintaining tolerance 89,90. These developments highlight CAR cell therapy's potential for both cancer and autoimmune diseases.

In summary, the combined application of CAR-NK cells and CAR-M cells brings new hope for the treatment of solid tumors. Future research should focus on optimizing their roles within the tumor microenvironment to overcome existing challenges and achieve more effective treatments. CAR cell therapy shows great promise, and with continuous technological advancements and deeper clinical trials, it is poised to bring new hope and potential cures to more cancer patients in the future.

## 3. Different transfection vectors

**Figure 4:**
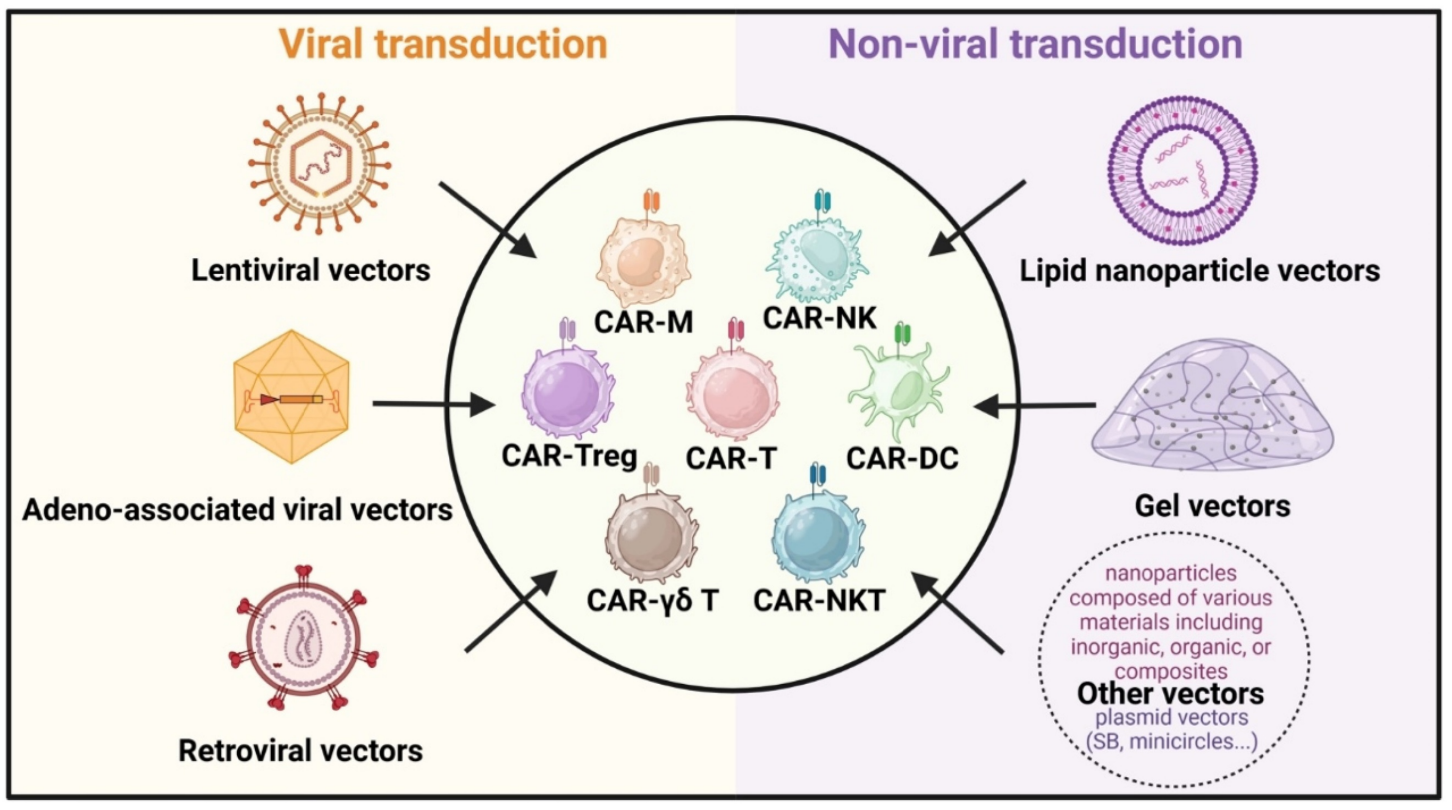
**Delivery strategies for *in vivo* CAR construction**. The primary vectors for viral transduction **(left)** include lentiviral vectors, adeno-associated viral vectors, and retroviral vectors. Non-viral transduction **(right)** vectors consist of lipid nanoparticle vectors, gel vectors, and other nanoparticle vectors like plasmid vectors (Sleeping Beauty, minicircles, etc.).

*In vivo* construction of CAR cells has become a pivotal area of interest in CAR cell therapy due to its rapid preparation time and cost-effectiveness. It enhances the survival rate and functional efficacy of CAR cells within the body, offering a promising future for immunotherapy. Further studies on various delivery platforms, each with its own unique set of benefits and challenges, could influence therapeutic effectiveness. Beyond traditional viral transduction methods like lentiviral, retroviral, and adeno-associated viral vectors, non-viral delivery strategies such as lipid nanoparticles and gels have emerged (**Figure 4**) 98. These innovative approaches are designed to improve the safety and efficacy of CAR cell therapy, opening new avenues for treating various tumors.

### 3.1. Lentiviral vectors and Retroviral vectors

**Figure 5:**
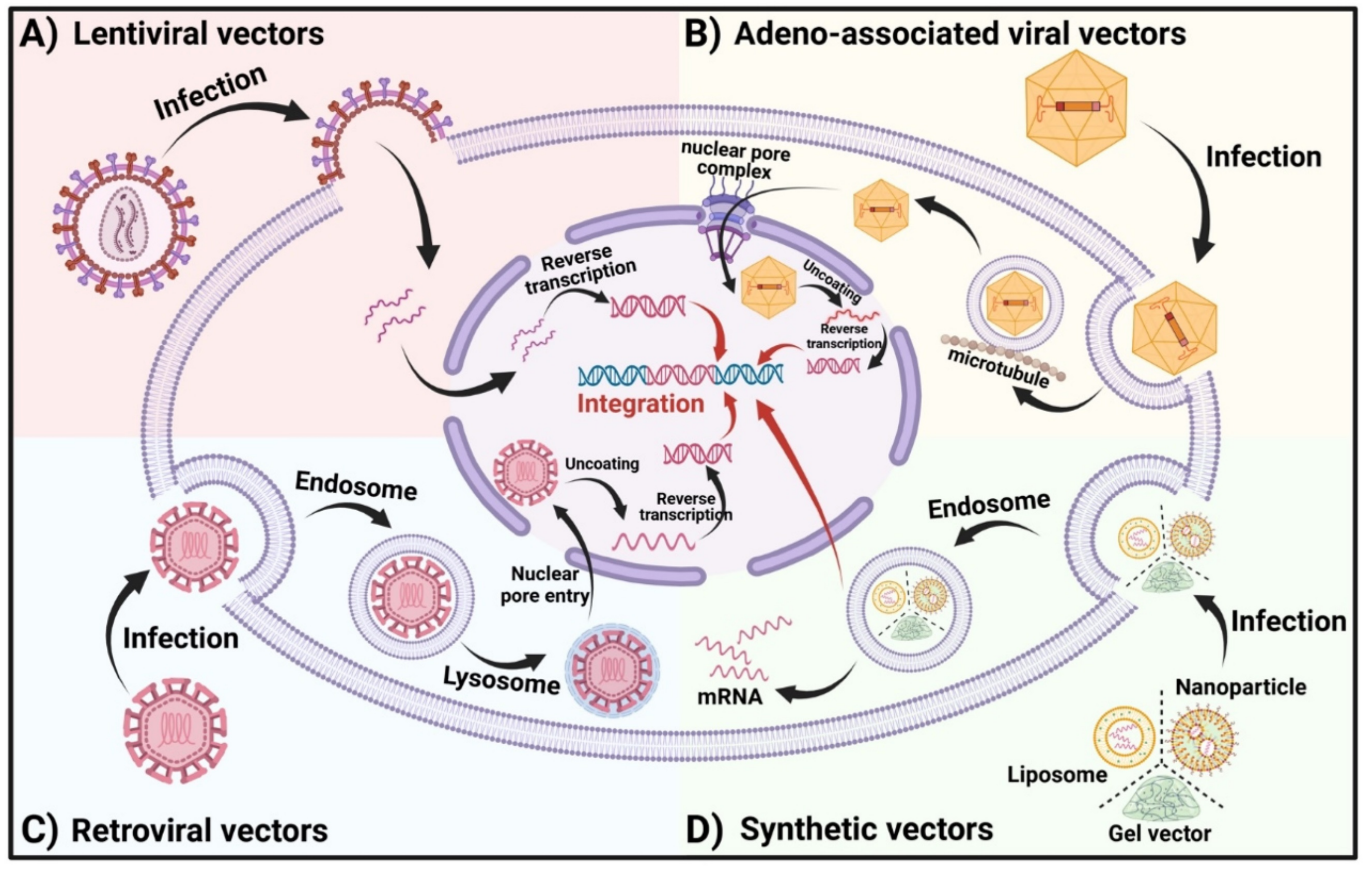
** The Vector entry modes into the cell.** (A) LVs contain one or more viral glycoproteins and two copies of a single-stranded RNA (ssRNA) genome encapsulated within a nucleocapsid. Once inside, the transferred gene undergoes reverse transcription, is transported into the nucleus, and integrates into the host genome. (B) After internalization, AAVs are enclosed in endocytosed vesicles. The acidification of these vesicles triggers viral escape into the cytoplasm, where the viruses utilize the cellular microtubule transport system to approach the nucleus. AAVs then interact with the nuclear pore complex to gain entry into the nucleus. Once inside, the single-stranded DNA undergoes conformational changes to form double-stranded DNA, which may subsequently integrate into the host genome. (C) Retroviruses bind to host cell surface receptors via their glycoproteins. Subsequent membrane fusion introduces the viral capsids into the cells. In the nucleus, the capsids uncoat, and the viral RNA is reverse transcribed and integrated into the host genome. (D) In synthetic vectors, CAR-encoding nucleic acids are complexed with NPs, LNPs, or gel-based carriers. After escaping the endosome, mRNA payloads are available for translation, while packaged DNA may reach the nucleus for potential integration into host chromatin.

Lentiviral (LV) transduction is widely used in conventional CAR cell engineering for the efficient delivery of CAR transgenes 99. Derived from the human immunodeficiency virus (HIV), these retroviruses have been engineered to remove virulence genes and incorporate exogenous target genes as classical LV vectors. LVs are typically pseudotyped with the glycoprotein from vesicular stomatitis virus (VSV-G), resulting in a diameter of 120-150 nm. VSV-G mediates cellular entry through the low-density lipoprotein receptor (LDLR) and its family members, which are expressed on many cell types 100. This class of pseudotyped viruses can integrate foreign genes into the host genome, ensuring stable expression and the capability to infect both dividing and non-dividing cells 101. Once inside the cell, the LV genome is reverse transcribed to DNA in the cytoplasm, forming a pre-integration complex that enters the nucleus, where the DNA integrates into the cellular genome (**Figure 5A**). LV vectors serve as a pivotal gene delivery method in *in vivo* CAR construction, introducing CAR-encoding genes into target cells to enable CAR expression. The primary advantage of LV vectors is their ability to achieve stable gene integration into the host genome, thus ensuring sustained gene expression 102. However, challenges such as potential pathogenicity and the risk of random genome integration, which could lead to gene mutations, remain. Of particular concern is the fact that several cases of leukemia have been reported by the FDA following the use of LV in CAR-T cell therapy. Consequently, the FDA mandates that patients be informed of these associated risks. Despite these risks, LV vectors are a promising option for gene and cell therapy 103. In 2018, 54% of CAR-T cell generation clinical studies in the US utilized LV vectors as carriers 104. The two market-leading CAR-T cell therapies, Kymriah and Yescarta, which target CD19, also employ LV as their vector system. Additionally, over 100 ongoing clinical trials utilizing LV, predominantly focusing on immunology, hematological diseases, and cancer, underline its broad potential applications 105. Pioneering work by Christian J. Buchholz *et al.* demonstrated the induction of CAR-T cells *in situ* in immunodeficient mice using second-generation anti-CD19-CAR gene-encapsulated LV, exhibiting anti-tumor activity 106. Samuel K. Lai *et al.* developed a bispecific conjugate directing LV vectors to T cells for specific *in vivo* engineering of CAR-T cells, showcasing that LV-modified *in vivo* CAR-T cells possessed antineoplastic activity 107. Kathryn R. Michels and her team developed an *in vivo* CAR T-cell engineering platform, VivoVec, based on a lentiviral vector. Their candidate drug, UB-VV100, employs surface-engineered lentiviral particles that can bind, activate, and transduce T cells, enabling these cells to express an anti-CD19 CAR and a rapamycin-activated cytokine receptor (RACR) system. Preclinical studies demonstrated that UB-VV100 successfully activated T cells, achieved CAR T-cell transduction, and effectively eliminated tumor cells. Additionally, *in vivo* studies on humanized mice and canine models showed that the biodistribution of UB-VV100 was largely confined to immune cells and did not induce significant tissue pathology. This research provides a novel *in vivo* approach to engineering CAR T cells, potentially simplifying the therapeutic process 108. Future research will likely focus on enhancing the safety and specificity of LV vectors, minimizing potential side effects, and optimizing vector design to meet diverse treatment needs. The continued utilization of LV in genetic engineering, particularly for the *in vivo* construction of chimeric antigen receptors, opens new avenues for gene and cellular therapies with profound clinical implications. As technology advances and research progresses, the application of LV as an *in vivo* construction vector is expected to expand, offering greater potential in the field of gene therapy.

Retroviruses (RVs) are a class of viruses characterized by single-stranded RNA genomes and their ability to reverse-transcribe RNA into DNA, mediated by the enzyme reverse transcriptase 109. Upon contact with a host cell, viral glycoproteins bind to cell surface receptors, facilitating membrane fusion and the entry of the viral capsid into the cell. Inside the host cell, reverse transcriptase synthesizes cDNA from the viral RNA, which is then transported into the nucleus and integrated into the host's chromosomal DNA with the assistance of integrase (**Figure 5C**). RVs are extensively utilized as gene delivery vectors in medicine due to their robust transduction capabilities across various cell types and relatively lower risk of integration compared to other vectors. A significant application of RVs includes the *in vivo* construction of chimeric antigen receptors (CARs). Despite their many advantages, RVs have some drawbacks, such as the potential for insertional mutagenesis, which could lead to genomic instability 110. Nevertheless, RVs have demonstrated considerable success in clinical settings; for instance, RV-based CAR-T therapies have been notably effective in treating leukemia and lymphoma 111,112. Furthermore, researchers from North Carolina State University and North Carolina University have developed an implant called MASTER, which employs modified RVs to transfer the CAR gene into T cells directly within the host, reprogramming them into CAR-T cells. This technique can generate and deploy CAR-T cells *in vivo* within just one day, significantly streamlining the production process 113.

Lentiviral vectors and retroviral vectors both belong to the retrovirus family and are capable of integrating exogenous genes into the host cell genome, enabling long-term stable expression. The primary differences between the two lie in their host range, gene integration sites, safety, and transduction efficiency. Lentiviral vectors can infect both dividing and non-dividing cells, with more random gene insertion and higher safety, making them widely applicable across various cell types. In contrast, retroviral vectors can only infect dividing cells, and their gene insertion tends to occur near promoter or enhancer regions, posing a higher risk of insertional mutagenesis, thus limiting their application.

As gene therapy technology continues to advance, RVs are expected to play an increasingly vital role as *in vivo* gene delivery mechanisms. They hold significant promise as key tools in biomedicine, paving new paths for treating genetic disorders, cancer, and various other diseases.

### 3.2. Adeno-associated viral vectors

Adeno-associated viruses (AAVs) are small, non-enveloped viruses with a single-stranded DNA genome, which have emerged as one of the preferred vectors in gene therapy due to their efficient transduction capabilities, favorable safety profile, and wide host cell range 114. Following cellular entry through internalization, AAVs are encased within endocytosed vesicles. The acidification of these vesicles facilitates viral escape into the cytoplasm. The viruses then utilize the cellular microtubule transport system to move close to the nucleus, where they interact with the nuclear pore complex to enter the nucleus 115,116. Once inside, the single-stranded DNA undergoes structural changes to become double-stranded DNA, which may integrate into the host genome (**Figure 5B**). The AAV genome comprises genes that code for specific antigen receptors, and these genes are engineered to facilitate the expression of the receptors upon the infection of host cells. This allows the modified cells to target and bind to specific antigens, offering novel strategies and approaches for cancer immunotherapy, viral therapy, and other disease treatments. A key advantage of AAV vectors is the existence of multiple serotypes, which exhibit different tissue tropisms, allowing for more precise targeting in gene therapy. For example, AAV9 has shown strong tropism for muscle and nervous tissues, making it a promising candidate for treating neuromuscular diseases, while AAV8 is often used for targeting the liver 117. This diversity enables the development of AAV-based therapies that can be tailored to specific tissue types or disease contexts. Strecker and colleagues developed a tumor-specific delivery system using an adeno-associated virus (AAV vector, HER2-AAVaPD-1, to deliver an anti-PD-1 immunoadhesin (aPD-1) directly to tumor cells. Additionally, they engineered HER2-targeted CAR-NK cells, specifically NK-92/5.28.z cells. When used in combination, these therapies significantly extended survival and controlled tumor growth in mouse models, without causing noticeable immune-related side effects. This dual approach offers a promising new strategy for the immunotherapy of glioblastoma 118. The utility of AAV was first demonstrated by R.J. Samulski *et al.*, who cloned the AAV genome into expression plasmids, laying the foundation for its application in gene therapy. When these plasmids were transfected into mammalian cell lines, they generated a substantial quantity of infectious viruses 119. Notably, Luxturna, an AAV2-based vector targeting the retinoid isomerase RPE65 gene associated with Leber congenital amaurosis and progressive blindness, became the second AAV-based therapy to gain commercial approval in 2017 120. Similarly, Zolgensma, an AAV-based treatment approved for spinal muscular atrophy (SMA), further demonstrates the clinical efficacy of AAV vectors 121. AAVs exhibit a lower risk of toxicity compared to other viral vectors. Despite the expanding use of AAV vectors in clinical applications, challenges remain in their large-scale production. High titers are required for effective *in vivo* delivery, and the production process can be labor-intensive and costly. Advances in manufacturing techniques, such as the development of optimized production platforms using suspension cell cultures and transient transfection methods, are being explored to address these limitations and make AAV therapies more accessible 122. In March 2021, Wu *et al.* reported that AAVs encoding third-generation CAR genes can efficiently reprogram immune effector cells to produce CAR-T cells *in vivo*, illustrating that AAVs can facilitate direct CAR-T cell formation within the body 123. In the context of CAR-T cell therapy, AAV vectors provide a novel platform for delivering CAR constructs directly into T cells *in vivo*, bypassing the need for *ex vivo* manipulation. This *in vivo* reprogramming strategy reduces the complexity and costs associated with traditional CAR-T cell production, potentially making CAR-T cell therapies more widely accessible. Nevertheless, like other gene delivery vectors, AAVs possess distinct advantages and limitations that require thorough evaluation 124. However, despite their low immunogenicity relative to other viral vectors, AAVs can still elicit immune responses, especially in patients with pre-existing immunity to the vector capsid proteins. Such immune responses can reduce the transduction efficiency and limit the efficacy of subsequent administrations, which is a significant challenge in clinical settings, particularly for therapies requiring long-term or repeated dosing 125. Strategies to mitigate these immune responses, such as the use of immunosuppressive drugs or modified AAV capsids, are being actively explored.

### 3.3. Lipid nanoparticle vectors

Liposomes have been recognized as a powerful medical tool for over five decades 126. Composed primarily of lipid molecules such as phospholipids, cholesterol, and surfactants, lipid nanoparticles (LNPs) form the core structure of liposomes 127. Phospholipids are the main components of LNPs, forming a bilayer membrane that encloses an internal aqueous phase. Cholesterol acts as a stabilizing and supportive element within the lipid bilayer, while surfactants on the LNP surface enhance stability and biocompatibility. These surfactants, which can be non-ionic, anionic, or cationic, are utilized to modulate the stability and charge of LNPs. The capability of LNPs to encapsulate drugs and deliver them precisely to targeted sites in the body highlights their immense potential for treating a wide range of diseases 128.

In recent years, LNPs have been employed to reprogram CAR cells *in vivo.* Margaret M. Billingsley and colleagues developed an *in vivo* CAR T cell engineering platform that employs ionizable LNPs conjugated with antibodies (Ab-LNPs) to target pan-T cell markers, facilitating efficient T cell transfection. This innovative approach enables the generation of functional CAR T cells within the body 129. Jonathan A. Epstein *et al.* developed a method to generate transient anti-cardiac fibrosis CAR T cells* in vivo* using targeted LNP delivery of modified mRNA 130. Jiang *et al.* designed a specialized LNP (CAR&Siglec-GΔITIMs LNP) that co-delivers CAR mRNA and truncated sialic-binding immunoglobulin-like lectin-G mRNA (Siglec-GΔITIMs mRNA). This LNP selectively adsorbs plasma proteins following intravenous injection and specifically edits liver macrophages to enhance phagocytosis and initiate immune responses within tumors, thereby effectively halting the progression of hepatocellular carcinoma 131. As a pivotal nanocarrier system, LNPs offer numerous advantages, including a simple structure, versatile compositions, and notable benefits 132-135. The efficiency of drug loading and targeted release is influenced by the adjustment of lipid composition and structure, which enhances drug bioavailability and efficacy. LNPs also exhibit excellent biocompatibility and biodegradability, rarely causing immune or toxic reactions. Despite the widespread use of LNPs in drug delivery, especially in gene therapy and vaccine development, their immunogenicity remains a concern. LNP formulations, particularly those used for mRNA vaccines, have been shown to trigger inflammatory responses. These immune responses can vary based on the composition of the lipids, with some ionizable lipids being more immunogenic than others 136. Modifying lipid structures or incorporating additional excipients to reduce immunogenicity is an ongoing area of research aimed at improving the clinical safety profile of LNP-based therapies 137. Targeting can be improved by modifying surfactants or lipid molecules, reducing drug toxicity to normal tissues, and enhancing therapeutic effectiveness. The size, shape, and surface properties of LNPs can be precisely tailored to meet various drug requirements through adjustments in formulation and processing conditions. Recent advancements in LNP technology have focused on the development of new lipid materials to enhance stability and functionality. For instance, the incorporation of ionizable lipids in LNPs has dramatically improved the efficiency of nucleic acid delivery by facilitating endosomal escape. Additionally, functionalizing the LNP surface with targeting ligands, such as peptides or antibodies, enables precise targeting of specific cell types, further enhancing therapeutic potential. PEGylation (the attachment of polyethylene glycol chains) is another widely used strategy to extend the circulation time of LNPs *in vivo* by reducing their recognition by the immune system 138. Furthermore, the preparation method for LNPs is straightforward and cost-effective, lay a foundation for large-scale production and clinical applications. The success of LNPs in clinical applications is best exemplified by their use in mRNA vaccines for COVID-19. Both Pfizer-BioNTech and Moderna's vaccines utilize LNPs to deliver mRNA encoding the spike protein of the SARS-CoV-2 virus, enabling the body's immune system to recognize and combat the virus. These vaccines have demonstrated the safety and efficacy of LNPs as drug carriers at a global scale, marking a significant milestone in the field of nanomedicine. This success has further fueled research into applying LNPs for a range of other therapies, including cancer treatments and genetic disorders 139.

However, LNPs face several challenges, including potential instability in the biological environment, which can lead to uneven drug release or reduced efficacy. Some LNPs may also induce cytotoxicity or immune responses, which can compromise clinical safety. Additionally, the drug loading capacity of LNPs is currently limited, potentially hindering the efficient delivery of certain drugs. Future advancements are anticipated to address these issues by enhancing stability, reducing toxicity, increasing drug loading capacity, and developing more functional nanoparticles. Improving LNPs to create a safer and more efficient drug delivery platform will drive further innovations and progress in clinical therapies.

### 3.4. Gel vectors

As water-based materials with a three-dimensional network structure, gels are highly recognized in the medical field for their exceptional biocompatibility and versatility as carriers for bioactive substances, particularly in immunotherapy 140,141. This biomaterial-based hydrogel is designed to encapsulate and deliver viral particles, enhancing their retention at the target site and enabling controlled release. The system aims to optimize the local delivery of viral vectors, ensuring efficient transfection and improving therapeutic outcomes. The utilization of gels in the* in vivo* construction of CAR-T cells, a pivotal strategy in immunotherapy, has gained substantial attention. Gels provide a scaffold that not only supports cellular structures but also prolongs the release duration of therapeutic agents 142. Hydrogels possess unique mechanical properties, including high water content and the ability to mimic the extracellular matrix, making them ideal for the controlled release of drugs and immune-modulating agents 143. Their viscoelastic nature enables them to conform to irregularly shaped surgical cavities, ensuring precise delivery of therapeutic agents in solid tumors, such as gliomas. Furthermore, the pore size of hydrogels can be fine-tuned to regulate the diffusion of bioactive molecules, offering an additional level of control in the therapeutic process 144. This capability is forging new paths in immunotherapy research and application, potentially revolutionizing cancer treatment. By constructing an injectable nanoparticle-hydrogel superstructure (NP-hydrogel superstructure), this study aims to *in situ* induce the generation of glioma stem cell (GSC)-specific CAR macrophages/microglia (MΦs) in the tumor resection cavity, thereby preventing the recurrence of glioblastoma (GBM) 145.

Wu *et al.* developed an innovative injectable supramolecular hydrogel system loaded with CAR plasmids to continuously modify CAR-T cells at solid tumor sites 146. Additionally, other researchers introduced a gene nanocarrier-injectable hydrogel superstructure, effectively employed in the immunotherapy of malignant glioma 145. In post-surgery glioma models, the hydrogel served as a 'filler' to deliver macrophage-targeted editing nanocarriers and CD47 antibodies into the tumor cavity. This approach aims to edit local macrophages to enhance their phagocytic activity against GSCs while blocking the tumor's “don't eat me” signals. This dual strategy activates the adaptive immune system to clear residual GSCs post-surgery and establish immune memory, thereby preventing glioma recurrence. As a delivery vector, gels offer numerous benefits: they act as three-dimensional scaffolds for drug loading, regulate cell microenvironments by releasing growth factors to enhance CAR activity and anti-tumor effects, and maintain excellent biocompatibility, supporting cell survival and function while minimizing immune rejection 147-150. Exploiting the unique properties of gels can significantly advance the construction of CAR cells for further immunotherapy. Despite their numerous benefits, the use of hydrogels in clinical settings presents several challenges. For instance, the rate of hydrogel degradation can vary significantly depending on the material composition, potentially leading to either premature release of therapeutic agents or insufficient breakdown to facilitate immune clearance 151. Additionally, achieving consistent mechanical strength and ensuring that hydrogels maintain their structural integrity under physiological conditions remains a key area of research 152. Overcoming these challenges will be critical in advancing the clinical application of hydrogels for CAR-based immunotherapy.

### 3.5. Other vectors

In addition to LVs, AAVs, RVs, LNPs, and gel-based vectors, a variety of other vectors are employed for *in vivo* CAR construction. Nanoparticles (NPs), defined by their nanoscale dimensions, can be synthesized from inorganic, organic, or composite materials. Their extensive specific surface area, coupled with distinctive physical and chemical properties, endows NPs with remarkable versatility in drug delivery and biological imaging. Furthermore, their degradability and cell-targeting capabilities render them particularly suitable for *in vivo* CAR construction. For instance, Matthias T. Stephan *et al.* developed biodegradable NPs capable of programming immune cells to recognize and destroy cancer cells *in vivo*. NPs-programmed T cells have demonstrated rapid clearance of cancer cells in leukemia mouse models, significantly improving their prognosis 153. The NPs are engineered to deliver a CAR gene, which specifically targets T cells through molecular markers. This interaction promotes the integration of the CAR gene into the nuclear genome of the T cells, subsequently enabling the cells to express CARs. This innovation allows CAR-T cells to function as a precise and readily deployable “drug” for cancer therapy 154. The advancement of suitable vectors for *in vivo* CAR construction plays an increasingly pivotal role in medical progress and patient well-being.

Plasmid vectors such as Sleeping Beauty (SB) and minicircles are emerging as valuable tools for non-viral gene delivery in CAR cell therapy, offering a safer and more cost-effective alternative to viral vectors like lentiviruses or retroviruses. The SB transposon system facilitates stable integration of CAR constructs into the genome with a reduced risk of insertional mutagenesis, making it a promising option for clinical applications. Similarly, minicircles, which are smaller plasmids lacking bacterial sequences, lower immunogenicity and enhance gene expression efficiency. These advantages make plasmid vectors particularly attractive for in vivo CAR generation, as they support more efficient and durable CAR cell production while mitigating safety concerns and reducing overall costs.

Inamdar *et al.* utilized a porous collagen scaffold-based implantable device to recruit host T cells *in situ* within tumors. By employing strategies such as CAR incorporation, this device facilitates the reprogramming and amplification of T cells for the treatment of solid tumors. Experimental results demonstrate that the device effectively enhances T cell tumor recognition capabilities, leading to tumor regression, thereby providing a novel approach for CAR-T cell therapy 155. “Drydux” is an innovative biomaterial scaffold designed for the rapid and efficient *in situ* generation of tumor-specific CAR-T cells. This scaffold enhances the *in vivo* retention, functionality, and durability of the cells, enabling sustained tumor remission across various animal tumor models. It holds the potential to revolutionize CAR-T cell therapy for solid tumors 156.

## 4. Key considerations for constructing CAR *in vivo*

CARs within the complex environment of the human body presents significant challenges, including navigating intricate blood circulation systems and interacting with diverse cell types. The success of CAR cell therapies depends on the meticulous selection of appropriate cell types and vectors, as well as the employment of effective construction methods. Factors influencing the *in vivo* construction of CARs include the choice of cell types, vector selection, and specific methodologies. Each of these elements is critical in determining the ultimate efficacy of CAR cell therapies in treating various diseases.

### 4.1. Cell types

Selecting suitable cell types for *in vivo* modification is crucial for the success of CAR cell therapies. A thorough understanding of the impact of various cell types on CAR construction necessitates considering factors such as cell abundance, distribution, phagocytic capacity, and modification potential. Accurate analysis can aid in selecting the most appropriate cell type to achieve the desired therapeutic effect. For instance, macrophages play a critical role in tumor invasion, metastasis, immunosuppression, and angiogenesis 157. Saar Gill *et al.* modified macrophages with HER2-targeted CARs, achieving a remarkable tumor-killing effect in mouse models 158. Specifically, HER2-CAR-M was found to convert immunosuppressive M2 macrophages into pro-inflammatory M1 macrophages, thereby enhancing the cytotoxic effects of T cells in the tumor microenvironment. Another notable example involves Kupffer cells (KCs), which constitute 80-90% of macrophages and are primary responders to foreign particles in the sinusoids of liver. Jiang *et al.* developed a novel strategy that transforms KCs into CAR-KCs for HCC treatment. LNPs were used to specifically target liver macrophages to deliver mRNA encoding CAR and a modified form of CD24-Siglec-G (Siglec-GΔITIMs) lacking ITIMs. This innovative approach enhanced the phagocytic activity of liver macrophages in HCC mouse models, significantly reducing tumor burden and improving survival rates. The appropriate choice of cell types facilitates CAR cell therapies, making them an effective and adaptable strategy for HCC treatment 128.

### 4.2. Construction methods

Construction methods, including gene editing technologies and transfection techniques, are also fundamental for the successful *in vivo* construction of CARs 159. Gene editing employs precise tools to modify, edit, or regulate an organism genome, facilitating the insertion, deletion, or alteration of specific genes. This approach effectively changes an organism's genetic traits 160. Prominent gene editing tools, such as CRISPR/Cas9, TALEN, and ZFN, enable targeted DNA modifications at specific genomic locations. These tools hold significant potential for treating genetic diseases, advancing biological research, and enhancing bioengineering efforts 161. Conversely, transfection technology involves the transfer of external DNA or RNA into target cells 162. Techniques such as viral, chemical, and electrical transfection are employed to introduce the CAR gene into cells, facilitating the creation of CAR cells.

Further enhancing the application of these gene editing tools, Jennifer R. Hamilton *et al.* demonstrated how these technologies can be tailored for precision targeting. By using antibody fragments displayed on membrane-derived particles encapsulating CRISPR-Cas9 proteins and guide RNAs, they successfully directed genome editing tools to specific cells 163. This antibody-targeted Cas9 delivery vector preferentially enables genome editing in homologous target cells within a mixed cell population *in vivo*. Additionally, Cas9-encapsulated delivery vectors (Cas9-EDVs) effectively generated genome-edited CAR-T cells in humanized mice. This approach underscores a programmable delivery modality with broad therapeutic potential, utilizing retroviral virus-like particle (VLP) assembly for transient delivery of Cas9 ribonucleoproteins (RNPs). More importantly, Cas9-EDVs achieved targeted genome editing in CAR-T cells without off-target effects on hepatocytes. These findings highlight the potential of EDVs as a programmable platform for delivering molecular cargo specifically to desired cell types for complex genome engineering *in vivo*. Understanding the variables that influence the *in vivo* construction of CARs is essential for optimizing the effectiveness of CAR cell therapy.

## 5. Other factors of construction of CAR

**Figure 6:**
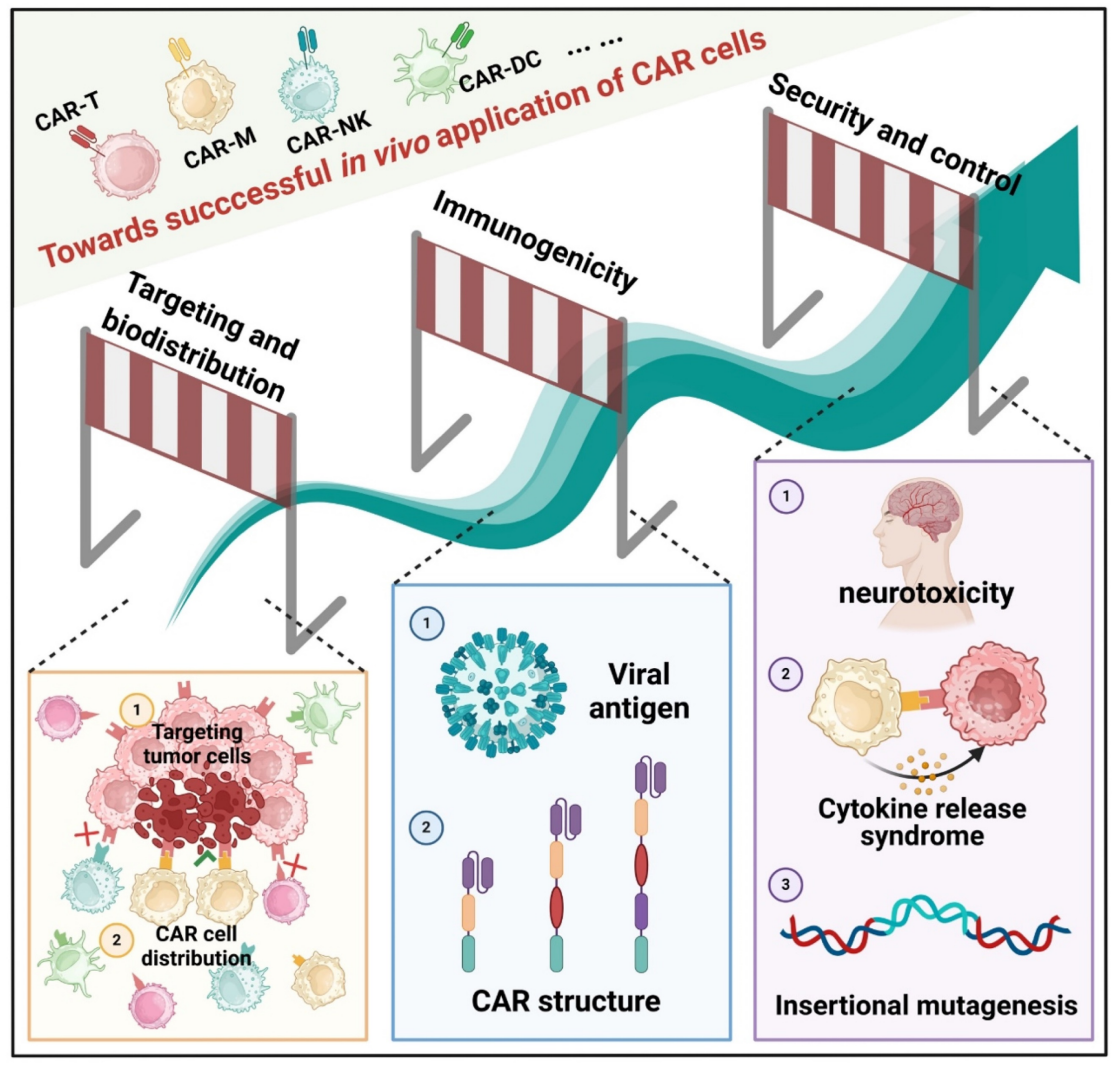
**Crucial factors to consider for the application of CAR cells.** 1) Targeting and biodistribution are crucial for optimizing therapeutic efficacy and advancing novel technologies. 2) CAR immunogenicity is a significant concern due to its diverse origins and multifaceted impact on treatment outcomes. 3) Safety and control-related challenges are of particular concern in *in vivo* CAR technology, including cytokine release syndrome (CRS), neurotoxicity, and potential insertional mutations during gene delivery. These may impair normal cellular function and induce tumorigenesis.

The *in vivo* application of CAR cells is a complex and challenging field that necessitates consideration of multiple critical factors. In the following sections, we will delve into issues related to the targeting and biodistribution of CAR cells, immunogenicity, and safety and control during *in vivo* application (**Figure 6**).

### 5.1. Targeting and biodistribution

CAR technology holds a significant role in tumor immunotherapy, with efficacy and safety critically hinging on precise targeting and effective biodistribution. The ability of CARs to target tumor cells fundamentally depends on their recognition of tumor-associated antigens 164. These antigens are typically categorized into two types: tumor-specific antigens, such as neoantigens arising from gene mutations, and tumor-associated antigens, such as CD19 and BCMA, which are predominantly overexpressed in tumor cells compared to normal cells 165. Optimizing CAR design by refining the antigen-binding region to increase affinity and specificity for tumor antigens can markedly enhance specificity 166. Additionally, employing bispecific or multispecific CAR structures to recognize multiple tumor antigens simultaneously mitigates the risk of off-target effects due to the heterogeneous expression of single antigens 167.

Once the target antigen is identified, the biodistribution of CAR cells within the body becomes crucial to treatment outcomes. Distribution is influenced by several factors, including the expression of cell-surface molecules, the tumor microenvironment, and immune system regulation 168,169. For instance, certain adhesion molecules enhance the interaction of CAR cells with vascular endothelial cells, facilitating their migration out of blood vessels and into tumor tissues. Additionally, chemokine receptors on CAR cells enable them to migrate towards chemokines secreted within the tumor microenvironment, significantly affecting CAR cell biodistribution 170. The tumor microenvironment is typically immunosuppressive, enriched with inhibitory cytokines, immunosuppressive cells, and extracellular matrix components, which can impede CAR cell infiltration and survival. For example, PD-L1 molecules highly expressed in tumor tissues can bind to PD-1 receptors on CAR cells, inhibiting their activation and proliferation 171. Moreover, the harsh conditions of low oxygen, limited nutrients, and acidic pH within tumor tissues can negatively impact the survival and function of CAR cells. To address these challenges, researchers have developed hypoxia-resistant CAR designs and metabolic reprogramming strategies 172.

The immune system plays a critical role in regulating the biodistribution of CAR cells 173. *In vivo*, CAR cells are frequently identified as foreign entities, subjected to immune surveillance and elimination. Additionally, the immune system can indirectly influence CAR cell distribution and function through cytokine secretion and immune cell activation. For instance, regulatory T cells can suppress CAR cell activation and proliferation by secreting inhibitory cytokines, while macrophages and dendritic cells can promote CAR cell clearance through antigen presentation. To enhance the biodistribution and therapeutic efficacy of CAR cells, various strategies have been implemented. Researchers have genetically engineered CAR cells to express molecules such as chemokine receptors and anti-apoptotic proteins, thereby improving their infiltration and survival 174. Additionally, combining CAR cell therapy with immunomodulators such as PD-1/PD-L1 inhibitors and CTLA-4 inhibitors can enhance the tumor microenvironment and boost CAR cell functionality 175,176. Innovative drug delivery systems, including nanotechnology and hydrogels, have been employed to improve CAR cell aggregation and retention at tumor sites. Investigating CAR cell targeting and biodistribution is crucial for optimizing existing therapies and developing safer, more effective next-generation CAR technologies. Future efforts aim to achieve more precise CAR targeting and improved biodistribution, ultimately enhancing survival outcomes for cancer patients.

### 5.2. Immunogenicity

When the CAR is introduced into the body, it is recognized as a foreign substance, triggering an immune response, also known as immunogenicity. CAR immunogenicity remains a significant concern, as it can affect both the efficacy and safety of the treatment 177. In the context of CAR cells, immunogenicity arises from several sources. Firstly, the structure of the CAR itself may be recognized as foreign by the immune system. CARs often contain antibody fragments from different species or synthetic peptides, which are not native to the human body and are likely to trigger an immune response 178. Secondly, viral vectors or other gene delivery systems may introduce new antigens, which can also provoke an immune response 179. Additionally, the large-scale expansion and persistence of CAR cells in the body may attract immune system attention 180. The impact of CAR immunogenicity is multifaceted. On one hand, the immune response could reduce the survival and functionality of CAR cells, thereby diminishing the treatment's effectiveness. For instance, antibodies produced in the patient might bind to the CAR, preventing it from interacting with tumor antigens and impairing its tumor-killing capability. On the other hand, a strong immune response can cause serious adverse effects, such as cytokine release syndrome and neurotoxicity, posing significant health risks to the patient.

Scientists are exploring various strategies to mitigate CAR immunogenicity. In CAR design, using humanized antibody fragments or optimized peptide sequences can help reduce the presence of foreign components 181. Selecting more appropriate gene delivery systems, such as low-immunogenic viral or non-viral vectors, can minimize the risk of immune responses 182. Additionally, modulating the patient's immune system with immunosuppressive drugs can partially inhibit the immune attack on CAR cells. With an enhanced understanding of CAR immunogenicity, more effective strategies will be developed to address this issue.

### 5.3. Management of adverse toxicities

The safety and control issues arising from CAR *in vivo* technology have garnered increasing attention 183. While immune responses are essential for CAR cell therapy to exert potent anti-tumor effects, they can also lead to serious adverse reactions, threatening patients' health and even their lives.

CRS is a common and serious complication of CAR cell therapy 184. When CAR cells interact with tumor cells, they can rapidly activate immune cells, leading to the release of a large number of cytokines such as interleukin-6 (IL-6) and interleukin-1 (IL-1) 185. Symptoms of CRS range from mild fever, fatigue, and myalgia to severe hypotension, respiratory failure, multiorgan dysfunction, and even life-threatening conditions. The severity of CRS is typically graded based on clinical symptoms and laboratory parameters. Neurotoxicity is another significant adverse reaction associated with CAR cell therapy 186. Although the exact mechanism is not fully understood, neurotoxicity may involve the direct effects of cytokines, immune cell infiltration into the nervous system, and endothelial cell dysfunction. Symptoms of neurotoxicity are diverse and can include headache, delirium, confusion, seizures, aphasia, and ataxia. In severe cases, neurotoxicity can lead to permanent neurological damage.

Another critical safety issue is the risk of insertional mutagenesis during gene delivery, which can compromise normal cellular function and potentially induce tumorigenesis 187. CAR genes are generally integrated into the cellular genome via viral vectors or alternative gene transfer methods. This stochastic insertion can disrupt adjacent genes, leading to aberrant function or gene inactivation 188,189. Furthermore, CAR genes can be inserted into regulatory regions of the T cell genome, such as promoters, enhancers, or insulators, thereby influencing gene expression and regulation. In rare instances, CAR genes may integrate into oncogenes or tumor suppressor genes, resulting in dysfunction and heightening the risk of tumor development or progression. To mitigate the risks associated with insertional mutagenesis, scientists have implemented various strategies, including the optimization of gene transfer techniques, such as enhancing promoters and enhancers, and refining CAR structures 190. They have also carefully selected appropriate vectors tailored to specific objectives, including lentiviral vectors, adeno-associated viral vectors, γ-retroviral vectors, mRNA vectors, and plasmid vectors 191. The selection of insertion sites has been meticulously conducted, favoring “safe harbor” sites such as the AAVS1 site, CCR5 site, and Rosa26 site 192. Moreover, rigorous quality control and testing of CAR-modified cells are crucial to ensuring their safety and efficacy.

In CAR cell therapy, despite its remarkable antitumor potential, functional exhaustion remains one of the primary obstacles limiting its clinical efficacy 193. To address this issue, researchers have proposed various strategies to enhance CAR cell persistence and antitumor activity. Optimizing CAR structure is a key aspect of these efforts, with modifications to the intracellular signaling domains and the incorporation of costimulatory molecules such as CD28 or 4-1BB significantly improving CAR cell functional resilience 194. Gene-editing technologies, including CRISPR/Cas9, have been widely employed to knock out exhaustion-related genes such as PD-1, LAG-3, and TIM-3 195, or to enhance CAR cell metabolic activity to reduce exhaustion in hostile tumor microenvironments 196. Additionally, the combined use of immune checkpoint inhibitors, such as PD-1/PD-L1 monoclonal antibodies, can block inhibitory signals and further prolong CAR cell efficacy 197. To address antigen escape, multi-targeted or bispecific CAR designs have emerged as key solutions, with attempts also being made to create dynamically regulated CAR systems that adjust expression in response to environmental cues 198. In improving the tumor microenvironment, pharmacological interventions and gene modification techniques have been employed to inhibit the function of immunosuppressive cells 199. Moreover, combination therapies, such as CAR cells with tumor vaccines, radiotherapy, or chemotherapy, have been utilized to further enhance efficacy and delay exhaustion 200. These multifaceted approaches provide a strong foundation for improving the therapeutic efficacy of CAR cells and driving advancements in this field.

## 6. Concluding remarks and future perspectives

**Figure 7:**
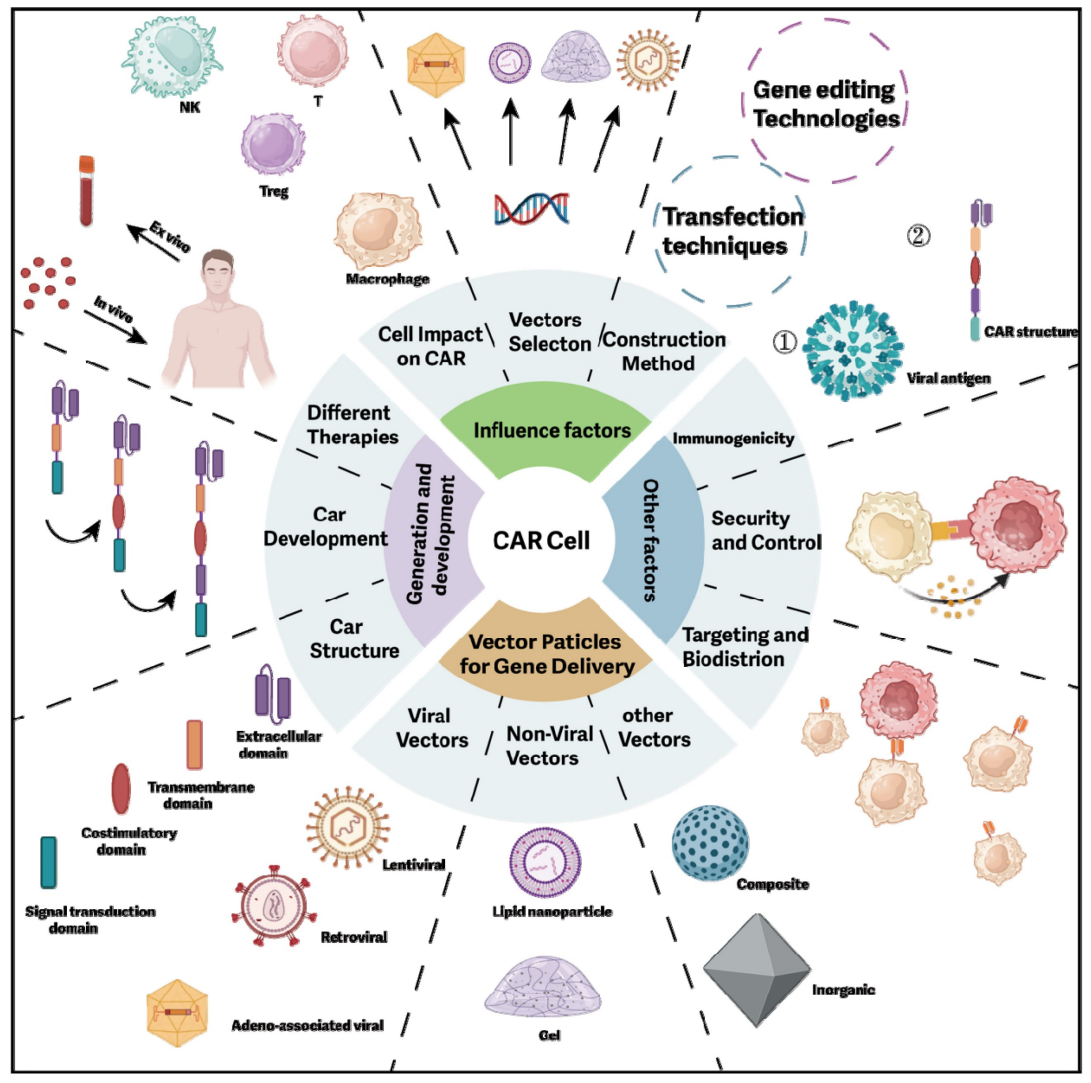
**The comprehensive conclusion for CAR cell construction *in vivo*.** This comprehensive summary covers the critical factors in constructing CAR cells, including gene editing, transfection techniques, vector selection, cell types, and CAR structure. It also illustrates the related elements of CAR cell construction and their interrelationships.

This article provides a comprehensive review of the CAR structure, its evolution, various forms, and delivery methods for CAR cells. Additionally, it examines the critical factors influencing CAR development, offering valuable insights for future research (**Figure 7**).

Selecting an appropriate vector for constructing CARs *in vivo* is paramount. A suitable vector facilitates the efficient transfer of the CAR gene into patients' immune cells, thereby enhancing the expression efficiency and stability of CAR cells. Continuous advancements in vector technology offer promising opportunities for the further development of CAR cell therapy. Beyond CAR-T cell therapy, CAR-NK, CAR-M, and other CAR cells represent emerging avenues for cancer treatment, demonstrating significant anti-tumor potential and undergoing extensive research and clinical trials.

However, traditional CAR preparation is complex and costly, limiting its widespread application. Several strategies are being developed to address these challenges: (1) Develop more efficient and cost-effective preparation technologies to streamline the process and reduce time and resource consumption. (2) Optimize cell collection methods to minimize individual differences, ensuring consistent quality and quantity of collected cells. (3) Improve the precision and efficiency of gene modification by exploring better conditions for cell expansion and genetic modification to enhance CAR cell functionality. (4) Transform the tumor microenvironment to promote CAR cell homing by understanding and modifying the tumor microenvironment. (5) Enhance gene delivery technology to improve *in vivo* delivery efficiency and specificity, and develop highly specific vectors and delivery systems. (6) Advance research on CAR construction factors by identifying more tumor-specific targets, better understanding signaling pathways, and designing optimal combinations.

To enhance the efficacy and safety of CAR cell therapy, researchers are exploring combination strategies with other treatment modalities. Taking CAR-T cell therapy as an example, the integration of immune checkpoint inhibitors (ICIs) such as PD-1 and PD-L1 monoclonal antibodies with CAR-T cells has been extensively studied. These inhibitors restore the ability of T cells to attack cancer cells, thereby improving the persistence and effectiveness of engineered cells within the tumor microenvironment. Clinical trials have demonstrated that in certain malignancies, such as glioblastoma and neuroblastoma, the combination of CAR-T cells with ICIs shows promising safety and efficacy, though results vary depending on the type of tumor. Moreover, cancer vaccines and oncolytic virus (OV) therapies are emerging as novel combination strategies that enhance CAR-T cell activation, proliferation, and persistence, thereby boosting their antitumor activity. Preclinical studies and early clinical trials indicate that cancer vaccines and oncolytic viruses can significantly improve CAR-T cell efficacy, particularly in solid tumors. Finally, monoclonal antibodies targeting cytokines, such as tocilizumab and anakinra, have shown efficacy in managing CAR-T cell-associated toxicities. These antibodies reduce the incidence of CRS and immune effector cell-associated neurotoxicity syndrome (ICANS) by inhibiting pro-inflammatory cytokines, thereby enhancing the safety of CAR-T cell therapy. Combining CAR-T cells with other treatment modalities, through multi-targeted and multi-mechanistic synergistic actions, holds the potential to overcome current efficacy and safety challenges, providing patients with more effective and durable treatment options.

Moving forward, researchers aim to develop multi-target CAR cell therapies, where CAR cells capable of recognizing multiple tumor antigens, such as bi-specific or multi-specific CAR constructs, could enhance therapeutic efficacy and reduce tumor immune evasion. Developing safer and more efficient vectors, such as viral vectors with higher specificity and lower immunogenicity, or novel non-viral vectors, is crucial for improving the efficiency and accuracy of gene delivery. For example, a delivery platform based on protein-lipid vehicles (PLVs) integrates the advantages of both viral and non-viral vectors for the safe and effective *in vivo* delivery of DNA and RNA. By incorporating the fusion-associated small transmembrane protein (FAST) derived from orthoreovirus into the lipid formulation, this platform achieves broad gene delivery capabilities while demonstrating low immunogenicity and favorable tolerability. Additionally, further research on personalized treatments is essential. For example, tailoring specific CAR cell therapies based on the patient's tumor characteristics, immune status, and genetic background can enhance the specificity and effectiveness of the treatment. Exploring combination therapies of CAR cell therapy with immune checkpoint inhibitors, chemotherapy, radiotherapy, and targeted therapies could also improve the overall efficacy of cancer treatment. Moreover, future research should focus on the immunogenicity of CAR cells to develop more effective strategies to reduce immunogenicity and enhance the safety and efficacy of the treatment. Ideally, the painstaking efforts made within the immune-oncology space will further advance CAR cell therapy for better cancer treatment outcomes and patient survival rates.

With ongoing advancements in gene editing and transfection technologies, *in vivo* CAR construction is poised to become a key method of personalized treatment, leading to revolutionary advancements in cancer and disease treatment. Future research should focus on exploring diverse methods of *in vivo* CAR construction to enhance treatment efficacy, minimize side effects, and extend benefits to a larger patient population. Furthermore, through continuous technological innovation and multidisciplinary collaboration, it is anticipated that the current challenges can be overcome, bringing more hope for a cure to cancer patients. With an in-depth understanding of tumor biology and the immune system, CAR cell therapy is expected to achieve breakthroughs in a broader range of cancer types, paving the way for the development of precision oncology.

## Figures and Tables

**Table 1:** Solid tumor targets of CAR-T

Target	Cancer type	Clinical trials NCT number
EpCAM	Malignant neoplasm of nasopharynx, colon, esophageal, pancreatic, prostate, gastric, liver, and breast cancer	NCT03013712, NCT02915445, NCT02729493, NCT02725125, NCT05028933, NCT04151186
PD-L1	Mesothelioma, colorectal, lung, and liver cancer	NCT03330834, NCT05089266, NCT04489862, NCT03672305, NCT03060343
HER2	Metastatic malignant neoplasm in the brain, GBM, ependymoma, glioma, sarcoma, and CNS tumor Gastric, breast, ovarian, bladder, head and neck, lung, esophageal, colorectal, pancreatic, and salivary gland cancer	NCT04903080, NCT04650451, NCT03696030, NCT03500991, NCT04995003, NCT04903080, NCT04511871, NCT03740256, NCT02442297, NCT03198052, NCT01109095, NCT00902044, NCT03423992, NCT03267173, NCT05681650
EGFR	Glioma, lung, liver, GC, and other EGFR positive advanced solid tumor	NCT05060796, NCT04153799, NCT03618381, NCT03182816, NCT04976218, NCT02331693, NCT03198052, NCT02873390, NCT02862028
B7-H3	GBM, sarcoma, melanoma, liver, lung, breast, pancreatic, lung, ovarian, and adrenocortical cancer	NCT05366179, NCT05241392, NCT04483778, NCT04385173, NCT04077866, NCT04897321, NCT04670068, NCT03198052, NCT05341492, NCT05143151, NCT04691713, NCT04864821, NCT05515185, NCT05323201, NCT05366179, NCT05241392, NCT04483778, NCT04385173, NCT04077866, NCT04897321, NCT04670068, NCT03198052, NCT05341492, NCT05143151, NCT04691713, NCT04864821, NCT05515185, NCT05323201
MSLN	Malignant pleural mesothelioma, pleural mesothelioma, glioma, lung, liver, ovarian, pancreatic, breast, cervical, colorectal, GC, and other MSLN-positive advanced solid tumors	NCT03356808, NCT03916679, NCT03799913, NCT03545815, NCT03323944, NCT03182803, NCT03054298, NCT03030001, NCT02930993, NCT05057715, NCT04577326, NCT03198052, NCT03814447, NCT03638193, NCT03615313, NCT02792114, NCT02706782, NCT02465983, NCT02159716, NCT01897415, NCT01583686, NCT03497819, NCT05373147, NCT04562298, NCT04503980, NCT04203459, NCT05166070, NCT05089266, NCT03267173, NCT04489862, NCT02959151, NCT03356795, NCT05623488, NCT05693844, NCT03941626
MUC1	Sarcoma, glioma, cervical, lung, esophageal, colorectal, gastric, liver, pancreatic, breast, and OC	NCT03706326, NCT03525782, NCT03179007, NCT02617134, NCT02587689, NCT04025216, NCT04020575, NCT03356808, NCT03356795, NCT03356782, NCT03267173, NCT03198052
ROR1	Breast and lung cancer	NCT05274451, NCT05748938, NCT05638828
GD2	Glioma, NB, sarcoma, embryonal tumor, melanoma, and cervical cancer	NCT05298995, NCT03721068, NCT03373097, NCT04099797, NCT02992210, NCT02107963, NCT01953900, NCT01822652, NCT00085930, NCT03356795, NCT03423992, NCT03356782, NCT03356808, NCT05437315, NCT05620342

CNS: Central nervous system; GBM: Glioblastoma; GC: Gastric cancer; NB: Neuroblastoma; OC: Ovarian cancer;

**Table 2:** Current FDA approvals of CAR-T cell therapies

Number	Name	Trade Name	Target	Approval date	Indication	Approved Countries	Clinical benefit	Generation
1	Axicabtagene ciloleucel	Yescarta	CD19	2017.10	Large B-cell lymphoma 88,91,92	USA	CR:54%	Second
				2021.6		China	/	
2	Brexucabtagene autoleucel	Tecartus	CD19	2020.7	Mantle cell lymphoma 93	USA	CR:67%	Second
3	Idecabtagene vicleucel	Abecma	BCMA	2021.3	Multiple myeloma 94	USA	CR:25%	Second
4	Tisagenlecleucel	Kymriah	CD19	2017.8	Acute Lymphoblastic Leukemia 95	USA	CR:>90%	Second
5	Lisocabtagene maraleucel	Breyanzi	CD19	2021.2	Large B-cell Lymphoma 96	USA	CR:54%	Second
6	Ciltacabtagene autoleucel	Carvykti	BCMA	2022.2	Multiple myeloma 97	USA	CR:78%	Third

**Table 3:** Current completed clinical trials for CAR-NK cell therapies

NCT Number	Study Title	Conditions	Interventions	Phases	Study Start Date	Completion Date
NCT03056339	Umbilical & cord blood (CB) derived CAR-engineered NK cells for B lymphoid malignancies	B-lymphoid malignancies| acute lymphocytic leukemia| chronic lymphocytic leukemia| non-hodgkin lymphoma	Drug: fludarabine| drug: cyclophosphamide| drug: mesna | biological: IC9/CAR.19/IL15-transduced CB-NK cells|drug: AP1903	phase1|phase2	2017.6	2023.6
NCT04538599	RD13-01 for patients with r/r CD7+ T/NK cell hematologic malignancies	Hematologic malignancies	Drug: RD13-01 cell infusion	phase1	2020.9	2021.11
NCT05563545	Anti-CD19 CAR-engineered NK cells in the treatment of relapsed/refractory acute lymphoblastic leukemia	Acute lymphoblastic leukemia	Biological: CAR-NK-CD19 cells	phase1	2022.7	2022.11

**Table 4:** CAR-M cell therapies based clinical trials

NCT Number	Product name	Manufacturer	Target	Indication	Phases	Study Start Date	Completion Date
NCT04660929	CT-0508+ Pembrolizumab	Carisma Therapeutics	HER2	HER2 overexpressing solid tumors	Phase1	2021/2/2	2024/12/31
NCT03608618	MCY-M11	MaxCyte	Mesothelin	Relapsed/refractory ovarian cancer and peritoneal mesothelioma	Phase1	2018/8/27	2021/8/24
NCT05138458	MT-101	Myeloid Therapeutics	CD5	Refractory or relapsed peripheral T-cell lymphomas	Phase1 Phase2	2021/12/15	2025/10/1
NCT06562647	SY001	Cell Origin Biotech (Hangzhou) Co., Ltd.	Mesothelin	Overexpressing solid tumors	NA	2023/4/12	2025/4/1
/	CT-1119	Carisma Therapeutics	HER2	Mesothelin-positive solid tumors	preclinical	/	/
/	CAR-iMAC	Cell Origin	EGFRvIII, GPC3	Hepatocellular carcinoma	preclinical	/	/

## Abbreviations

AAV: adeno-associated virus
Ab-LNPs: lipid nanoparticles conjugated with antibodies
ADCC: antibody-dependent cellular cytotoxicity
AI: artificial intelligence
AML: acute myeloid leukemia
aPD-1: anti-PD-1 immunoadhesin
BCMA: B-cell maturation antigen
B7-H3: B7 homolog 3 protein
CAAR-T: Chimeric Autoantibody Receptor T
CAR: Chimeric antigen receptor
CAR-NKT: Chimeric Antigen Receptor Natural Killer T
CAR-T: Chimeric Antigen Receptor T
Cas9-EDVs: Cas9-encapsulated delivery vectors
CB: cord blood
CEA: carcinoembryonic antigen
CNS: Central nervous system
CRS: cytokine release syndrome
CSCs: cancer stem cells
DC: dendritic cell
ECM: extracellular matrix
EGFR: epidermal growth factor receptor
EpCAM: Epithelial cell adhesion molecule
FasL: Fas ligand
FAST: fusion-associated small transmembrane protein
GBM: glioblastoma
GC: Gastric cancer
GD2: disialoganglioside
GM-CSF: granulocyte-macrophage colony-stimulating factor
GSC: glioma stem cell
GvHD: graft-versus-host disease
HCC: hepatocellular carcinoma
HER2: human epidermal growth factor receptor 2
HIV: human immunodeficiency virus
HLA: Human Leukocyte Antigen
hPSC: human pluripotent stem cell
IBD: inflammatory bowel disease
ICANS: immune effector cell-associated neurotoxicity syndrome
ICIs: immune checkpoint inhibitors
IFN-γ: interferon-gamma
IgG: immunoglobulin G
IL-1: interleukin-1
IL-6: interleukin-6
IL-15: interleukin 15
iPSCs: induced pluripotent stem cells
ITAM: immune receptor tyrosine activation motif
KCs: Kupffer cells
KIRs: killer immunoglobulin-like receptors
LDLR: low-density lipoprotein receptor
LNPs: lipid nanoparticles
LV: Lentiviral
MICA/B: MHC class I chain-related protein A/B
MMPs: matrix metalloproteinases
MSLN: Mesothelin
MUC1: Mucin 1
MΦs: macrophages/microglia
NB: Neuroblastoma
NK: natural killer
NKRs: natural killer receptors
NP-hydrogel superstructure: nanoparticle-hydrogel superstructure
NPs: Nanoparticles
OC: Ovarian cancer
OV: oncolytic virus
PD-L1: Programmed cell death 1 ligand 1
PLVs: protein-lipid vehicles
PMN-MDSCs: polymorphonuclear myeloid-derived suppressor cells
RACR: rapamycin-activated cytokine receptor
RNPs: ribonucleoproteins
ROR1: receptor tyrosine kinase-like orphan receptor 1
RVs: Retroviruses
SB: Sleeping Beauty
scFv: single-chain variable fragment
SMA: spinal muscular atrophy
ssDNA: single-stranded DNA
ssRNA: single-stranded RNA
TCRs: T cell receptors
TIL: tumor-infiltrating lymphocytes
TME: tumor microenvironment
TRAIL: TNF-related apoptosis-inducing ligand
Tregs: regulatory T cells
TRUCK: T cells Redirected for Universal Cytokine Killing
VH: variable heavy
VL: variable light
VLP: virus-like particle
VSV-G: glycoprotein from vesicular stomatitis virus
